# Evaluating the clinical significance of SHMT2 and its co-expressed gene in human kidney cancer

**DOI:** 10.1186/s40659-020-00314-2

**Published:** 2020-10-16

**Authors:** Huan Wang, Tie Chong, Bo-Yong Li, Xiao-San Chen, Wen-Bo Zhen

**Affiliations:** 1 Department of Urology, Second Affiliated Hospital of Medical School, Xi’an Jiaotong University, Xi’an, Shaanxi 710061 P.R. China; 2 Urology Department, The First Hospital of Yueyang City, Yueyang City, Hunan Province 414000 China; 3 Female Urologic Institution, The First Hospital of Yueyang City, Yueyang City, Hunan Province 414000 China

**Keywords:** SHMT2, NDUFA4L2, Prognosis, Kidney cancer, Bioinformatic analysis

## Abstract

**Background:**

Kidney cancer is one of the most common cancers in the world. It is necessary to clarify its underlying mechanism and find its prognostic biomarkers. Current studies showed that SHMT2 may be participated in several kinds of cancer.

**Methods:**

Our studies investigated the expression of SHMT2 in kidney cancer by Oncomine, Human Protein Atlas database and ULCAN database. Meanwhile, we found its co-expression gene by cBioPortal online tool and validated their relationship in A498 and ACHN cells by cell transfection, western blot and qRT-PCR. Besides these, we also explored their prognostic values via the Kaplan–Meier plotter database in different types of kidney cancer patients.

**Results:**

SHMT2 was found to be increased in 7 kidney cancer datasets, compared to normal renal tissues. For the cancer stages, ages and races, there existed significant difference in the expression of SHMT2 among different groups by mining of the UALCAN database. High SHMT2 expression is associated with poor overall survival in patients with kidney cancer. Among all co-expressed genes, NDUFA4L2 and SHMT2 had a high co-expression efficient. SHMT2 overexpression led to the increased expression of NDUFA4L2 at both mRNA and protein levels. Like SHMT2, overexpressed NDUFA4L2 also was associated with worse overall survival in patients with kidney cancer.

**Conclusion:**

Based on above results, overexpressed SHMT2 and its co-expressed gene NDUFA4L2 were all correlated with the prognosis in kidney cancer. The present study might be benefit for better understanding the clinical significance of SHMT2 and provided a potential therapeutic target for kidney cancer in future.

## Background

Kidney cancer, as one of the most common cancers in the world, is becoming a severe global burden but lacks public attention [1]. Among various types of kidney cancer, clear cell renal cell carcinoma (ccRCC) was the most common subtype of kidney cancer, accounting for more than 85% of all kidney cancer [2]. Despite great achievement in diagnosis and therapy, because of the characteristics of high metastasis risk and poor response to radiotherapy and chemotherapy, the most of patients with advanced kidney cancer are still difficult to be cured and prolong the survival time [3]. Therefore, it is necessary to clarify the underling mechanism and find several clinically effective diagnostic and prognostic biomarkers to prevent its occurrence and re-occurrence.

Serine Hydroxymethyltransferase 2 (SHMT2), a protein coding gene, regulate glycine production in mitochondria, which is an essential intermediate for purine biosynthesis [4, 5]. In general, this gene is over-expressed in liver, lymph node and peripheral blood mononuclear cells. Nevertheless, it has been reported to a valuable marker in several cancers, including intrahepatic cholangiocarcinoma [6], large B cell lymphoma [7] and breast cancer [8]. Besides these studies, the mitochondrial one-carbon metabolic pathway involving in SHMT2 is also associated with patient survival in pancreatic cancer and gastrointestinal cancer [9, 10]. However, current studies have not focused on the relationship between SHMT2 and kidney cancer. And its expression profile and clinical significance of SHMT2 in human kidney cancer have not yet been investigated.

In our studies, we tried to explore the expression and prognosis of SHMT2 and its co-expression gene in kidney cancer for the first time by using multiple bioinformatics database. After experimental validation, we hoped that the assessment of both SHMT2 and its co-expressed gene in kidney cancer will be helpful in predicting kidney cancer prognosis and providing a novelty therapeutic target for individual treatment of kidney cancer.

## Materials and methods

### Ethics statement

No human or animal specimens were utilized in our studies. And all the datasets were acquired from the public databases and articles, which all met with the Declaration of Helsinki.

### Oncomine database

Oncomine database (https://www.oncomine.org/), a cancer database bioinformatics tool and online data-mining platform, was used to explore the mRNA levels of SHMT2 and NADH dehydrogenase [ubiquinone] 1 alpha sub complex, 4-like 2 (NDUFA4L2) in kidney cancer. We searched the database for the fold changes of SHMT2 and NDUFA4L2 in kidney cancer using the filters of differential analysis (cancer vs normal), cancer type (kidney cancer), sample type (clinical specimen), data type (mRNA), and gene (SHMT2 and NDUFA4L2). Students’ t-test was used to generate a p value. To obtain the most significant SHMT2 and NDUFA4L2probes, we set the following parameters: the cut-off of p value and fold change were defined as 0.01 and 2, respectively.

### GEPIA database

Gene Expression Profiling Interactive Analysis (GEPIA) (http://gepia.cancer-pku.cn) database, is a newly developed interactive web server developed by Zefang Tang, Chenwei Li and Boxi Kang of Zhang Lab, Peking University, focused on scrutinizing the RNA sequencing expression data of 9,736 tumors and 8,587 normal samples from the TCGA and the GTEx projects, using a standard processing pipeline. In this study, we used GEPIA to validate the expression levels of SHMT2. We set the following parameters: the cut-off of p value and fold change were defined as 0.01 and 2, respectively.

### Human Protein Atlas database

Human Protein Atlas (http://www.proteinatlas.org) is a Swedish-based program initiated in 2003 with the aim to map all the human proteins in cells, tissues and organs using integration of various omics technologies. In our study, the Human Protein Atlas was used for immunohistochemistry (IHC) validation of several candidate genes SHMT2 and NDUFA4L2.

### UALCAN database

The UALCAN web portal (ualcan.path.uab.edu/) has an important feature that aids querying based on the gene class. Meanwhile, it is a user-friendly, interactive web resource for analyzing cancer transcriptome data. Using UALCAN database, we analyzed the expression profiles of SHMT2 and NDUFA4L2 in normal and renal clear cell carcinoma samples based on clinicopathologic parameters, such as cancer stage, age, race, and tumor grade.

### cBioPortal database

The cBioPortal (www.cbioportal.org/) for Cancer Genomics was originally developed at Memorial Sloan Kettering Cancer Center (MSK), which contained both sequencing and pathological data on 30 different cancers. The kidney cancer (TCGA, renal clear cell carcinoma; Provisional) dataset including data from 538 samples with pathology reports was selected for further analyses of SHMT2.

### Cell culture and transfection

Human renal carcinoma cell lines A498 and ACHN cells were purchased from American Type Culture Collection (ATCC, Manassas, VA, USA). The cells were cultured in minimum essential medium (MEM, Hyclone) supplemented with 10% fetal bovine serum (FBS). All cells were maintained in a humidified incubator with 5% CO_2_ at 37 °C. Human SHMT2 cDNA ORF clone (cat. RC204239) and the empty control pCMV6-AC (cat. PS100020) were obtained from OriGene (Rockville, USA). A498 and ACHN cells were transfected with the expression vector or empty control using Lipofectamine 3000 (Life Technologies, CA, USA).

### qRT-PCR

Total cell RNAs were extracted utilizing the Trizol Reagent (Invitrogen, CA, USA). Then, the RNA samples were reverse-transcribed using the iScript cDNA Synthesis kit (Bio-Rad, CA, USA) following the manufacturer’s protocol. The primers of reference gene (β-actin) and NDUFA4L2 were designed by Primer5 software and synthesized by Shanghai Shenggong Bioengineering after BLAST comparison. Then, NDUFA4L2 mRNA was measured using qRT-PCR analysis with the following primers (sense:, 5′-TAATACGACTCACTATAGGG-3′ and antisense: 5′- TAGAAGGCACAGTCGAGG -3′). The reference gene is β-actin, sense: 5′-TTGTAACCAACT GGGACGATATGG-3′, and antisense: 5′-GATCTTGATCTTCATGGTGCTAG-3′ And the real-time RT-PCR assays were performed using the 7500 Fast Real-Time PCR System for quantitative mRNA detection and with iTaq Fast SYBR Green Supermix (Bio-Rad, Hercules, CA). The expression of genes in all groups was calculated using the 2-ΔΔCt method. All experiments were repeated three times independently.

### Western blot

Total proteins were separated by SDS-PAGE after denaturation and transferred onto polyvinylidenedifluoride (PVDF) membranes. After blocking with 5% skim milk, the membranes were incubated with rabbit anti-mouse monoclonal antibodies against. SHMT2 (1:300; Abcam, Cambridge, UK), NDUFA4L2 (1:1,000, Abcam, Cambridge, UK) and GAPDH(1:1000, Abcam, Cambridge, UK) overnight at 4 °C with shaking. Then, the membranes were washed in TBST. After that the membranes were washed and incubated with secondary antibody anti-rabbit IgG (1:2000, Santa Cruz, CA, USA) for 1.5 h at room temperature. Data obtained from the western blot experiments were analysed by Bio-Rad Quantity One 1D Analysis software (Bio-Rad, Hercules, CA, USA).

### Statistical analysis

Data from western blot and qRT-PCR were presented in the form of mean ± standard deviation (SD), and each experiment was independently repeated ≥ 3 times. Statistical analyses were performed using Graphpad Prism 5 (Graphpad software Inc, San Diego, CA, USA). Data were analyzed for statistical significance by Student’s t-test. p-value < 0.05 was considered statistically significant.

## Results

### Expression levels of SHMT2 in patients with kidney cancer

**Fig. 1:**
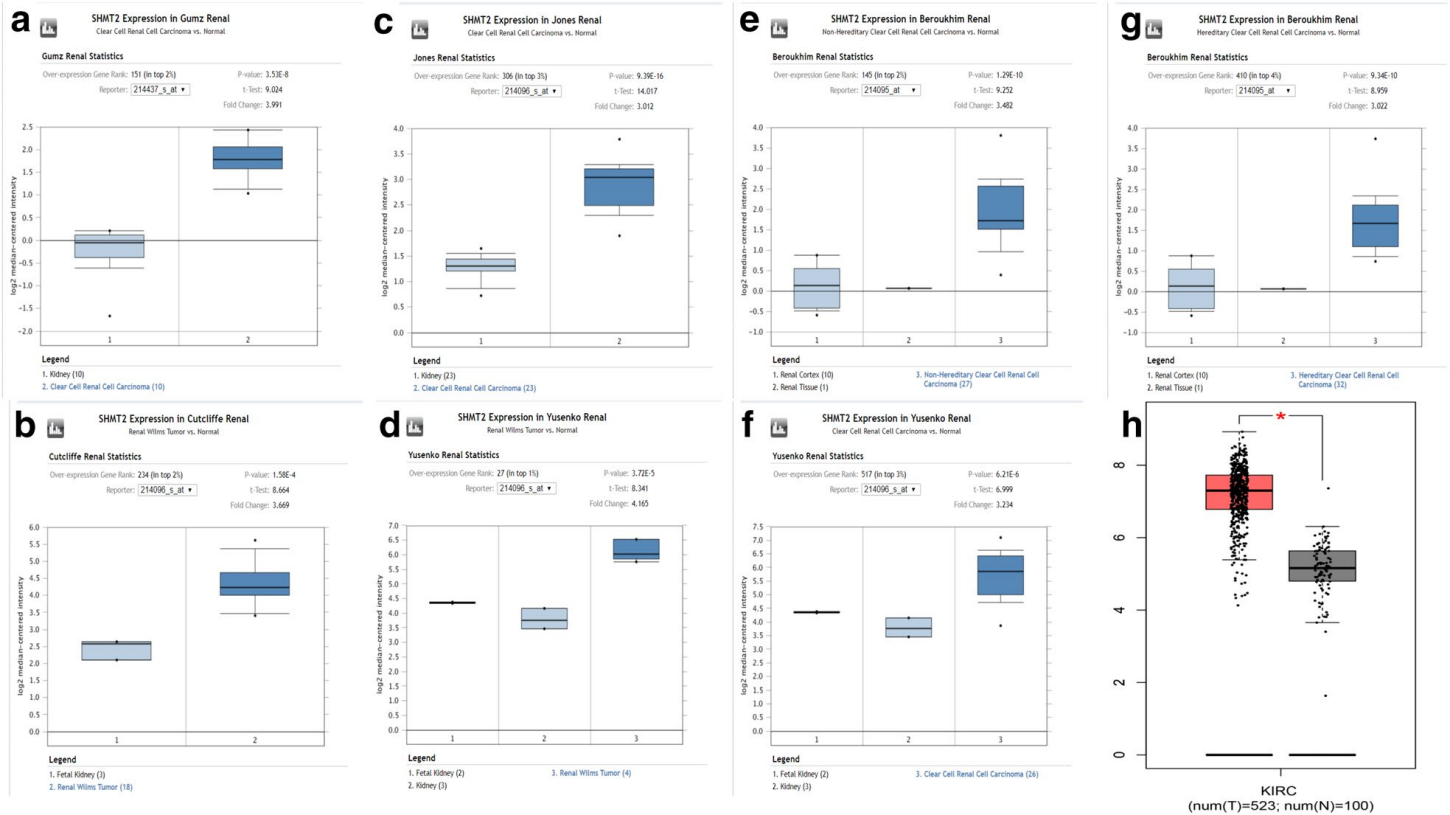
the expression level of SHMT2 mRNA in human different types of kidney cancer **a-g** The expression of SHMT2 in different types of kidney cancer datasets from Oncomine database; **h** The expression of SHMT2 in renal clear cell carcinoma patients from GEPIA database. A total of 523 renal clear cell carcinoma tissues (red bar) and 100 normal renal tissues (gray bar) were collected in this database

Utilizing the Oncomine database, we discovered the expression levels of SHMT2 in different kinds of kidney cancer with those in control samples. SHMT2 was up-regulated in 7 various kidney cancer datasets, compared to normol tissues (P‑value < 0.05 and Fold change > 2) including Renal Wilms Tumor [11], Clear Cell Renal Cell Carcinoma [11–13], Non-Hereditary Clear Cell Renal Cell Carcinoma [14] and Hereditary Clear Cell Renal Cell Carcinoma [14] (Fig. 1a-g). The comparisons of mRNA levels of SHMT2 in kidney cancer and healthy samples in each individual dataset were performed by using the Student’s t-test. In addition, we also confirmed that mRNA expression levels of SHMT2 was significantly increased in renal clear cell carcinoma tissues than in normal renal tissues, which further verified the results from GEPIA database (Fig. 1h). A total of 523 renal clear cell carcinoma tissues (red bar) and 100 normal renal tissues (gray bar) were collected in this database.

### Association of SHMT2 expression with clinicopathological features from patients with renal clear cell carcinoma

**Fig. 2:**
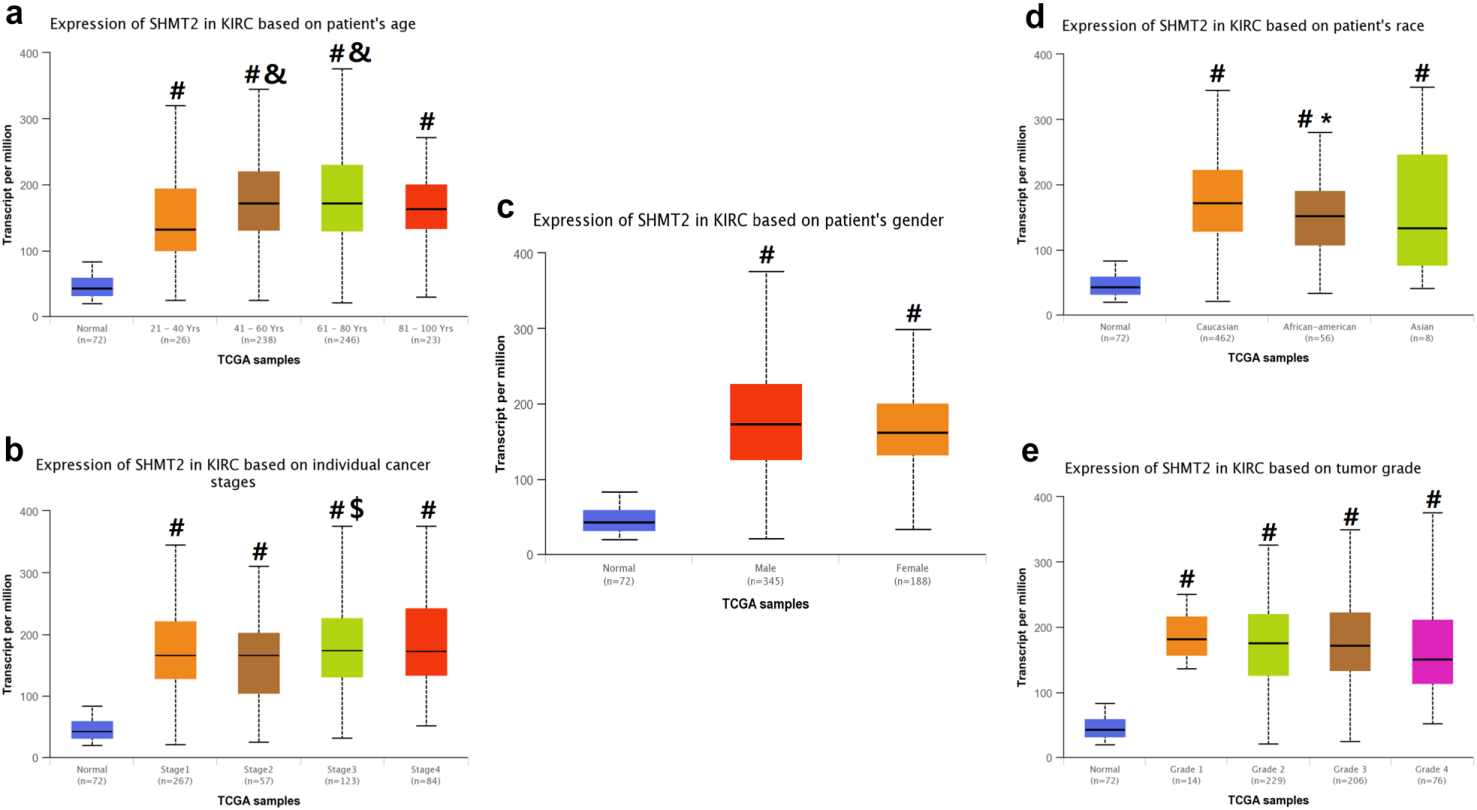
Association between SHMT2 gene expression and clinical pathological parameters in patients with renal clear cell carcinoma (UALCAN). **a** expression of SHMT2 in normal and renal clear cell carcinoma tissues based on patients’ age; **b** expression of SHMT2 in normal and renal clear cell carcinoma tissues based on cancer stages; **c** expression of SHMT2 in normal and renal clear cell carcinoma tissues based on patients’ gender; **d** expression of SHMT2 in normal and renal clear cell carcinoma tissues based on patients’ race; **e** expression of SHMT2 in normal and renal clear cell carcinoma tissues based on tumor grade. #P:compared to normal group; &P: compared to the age (21–40 years) group; $P: compared to the Stage 2 group; *P: compared to the Caucasian group

As shown in Fig. 2, we explored the expression of SHMT2 in normal and renal clear cell carcinoma tissues based on clinicopathologic parameters, such as cancer stage, age, race, and tumor grade by using UALCAN database. Our results indicated that SHMT2 was overexpressed in age (41–60 years) and (61–80 years) group compared to age (21–40 years) group (P < 0.05). In term of the cancer stages, SHMT2 was higher in Stage 3 compared to the Stage 2 (P < 0.05). And for races, the expression level of SHMT2 was higher in Caucasian patients compared to the African-American patients (P < 0.05). In regard to the tumor grade and patients’ gender, there existed no significant difference in the expression of SHMT2 among different groups (Fig. 2).

### High SHMT2 expression is associated with poor OS of patients with kidney cancer

**Fig. 3:**
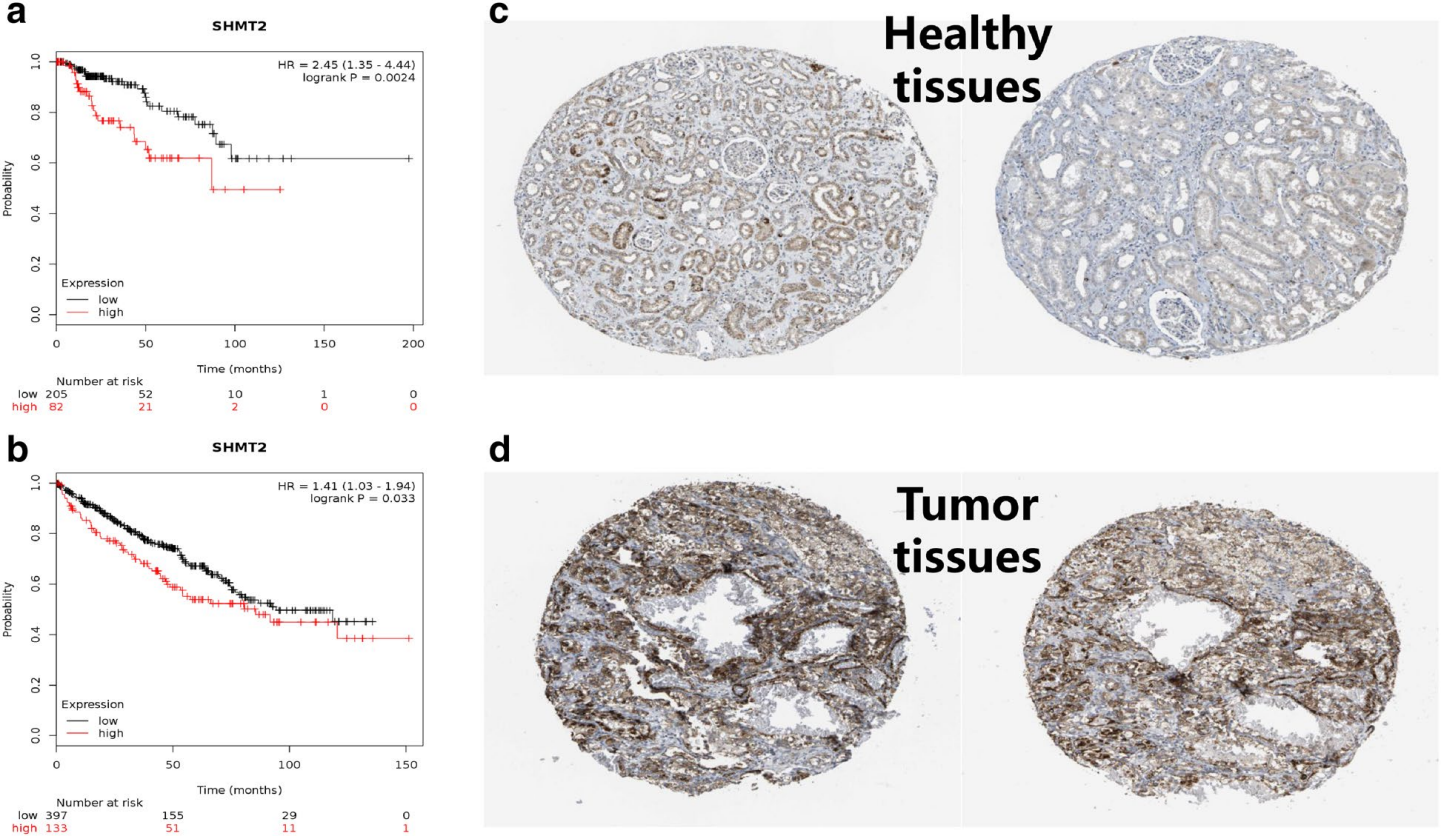
The prognostic value of SHMT2 mRNA in kidney cancer patients (K-M plotter) and the IHC of SHMT2 in kidney cancer (HPA). **a, b** Survival curve of SHMT2 for renal papillary cell carcinoma and renal clear cell carcinoma, respectively. **c, d**: IHC of SHMT2 in kidney cancer from healthy and pathological tissues

Using the Kaplan–Meier plotter, the prognostic values of SHMT2 in various kidney cancers were predicted. As shown in Fig. 3b, we found that overexpressed SHMT2 is associated with worse overall survival (OS) patients with renal clear cell carcinoma, with P = 0.033 and HR = 1.41 (1.03–1.94). For patients with renal papillary cell carcinoma, we found that high expressedSHMT2 are also correlated with poor overall survival compared to those with low expression, with P = 0.0024, HR = 2.45 (1.35–4.44), using the median as the cutoff value (Fig. 3a). Subsequently, to confirm the predictive results, we investigated the IHC pictures of kidney cancer by using the HPA database. The IHC pictures validated that the SHMT2 showed a strongly intensity in kidney cancers compared with those in healthy samples (Fig. 3c, d).

### Analysis of SHMT2′s co-expressed genes in kidney cancer

**Fig. 4:**
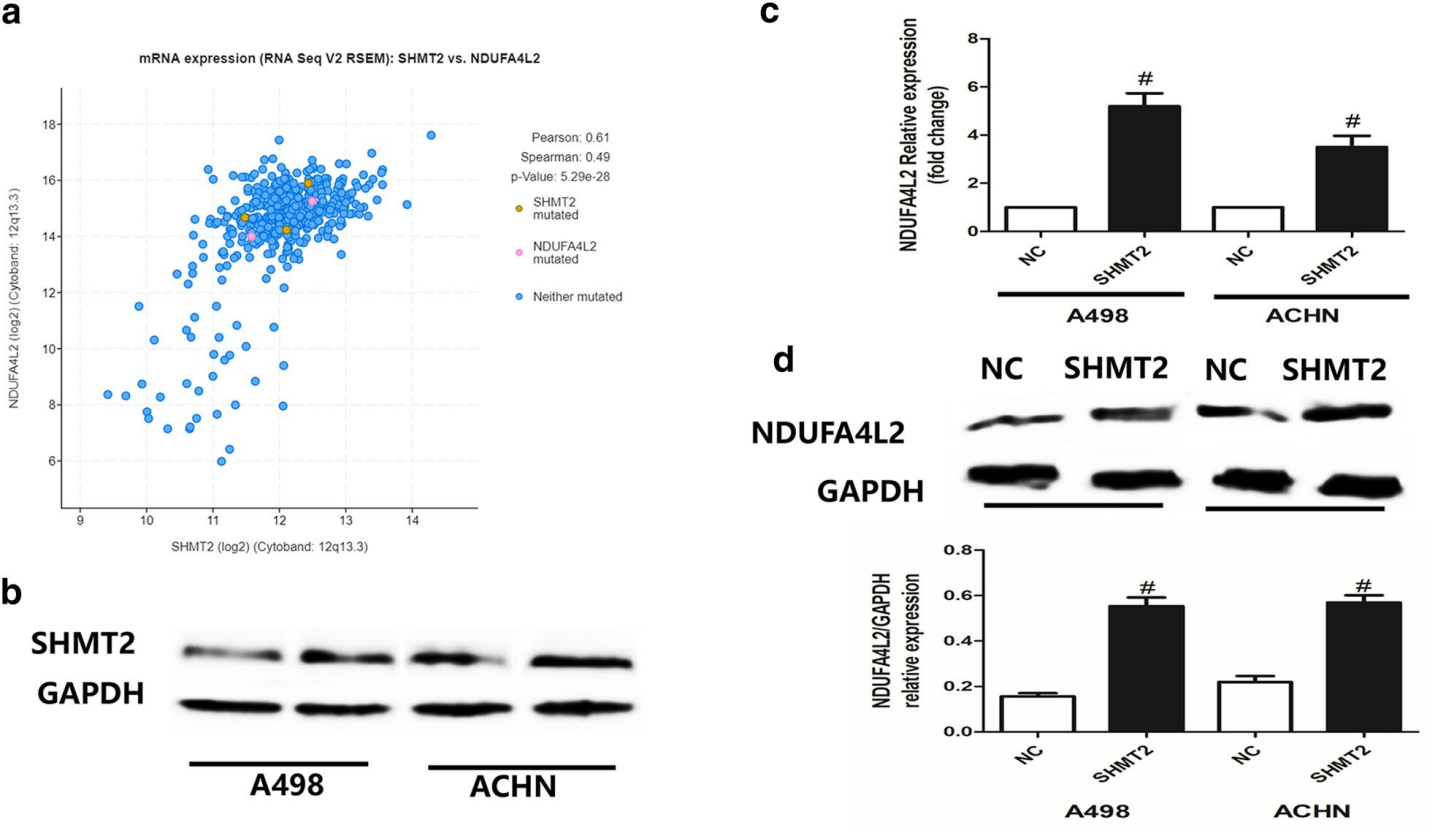
Analysis of SHMT2’s co-expressed genes in kidney cancer **a** regression analysis of the expression between SHMT2 and NDUFA4L2 in kidney cancer by using cBioPortal tool. **b** Western blot analysis and representative image of SHMT2 expression in A498 and ACHN cells 24 h after transfection of SHMT2 expression vector or the negative control. **c** NDUFA4L2 expression at mRNA level in A498 and ACHN cells 24 h after transfection of SHMT2 expression vector or the negative control. **d** Western blot assay and representative results of NDUFA4L2 expression at protein level in A498 and ACHN cells 24 h after transfection of SHMT2 expression vector or the negative control. n = 3; #p < 0.05, vs negative control

By examining the microarray data from cBioPortal online database for renal clear cell carcinoma (TCGA, Provisional), we identified the top 10 genes with positive correlation to SHMT2 expression in renal clear cell carcinoma, including NDUFA4L2, TCEA3, RACK1, HYI, LRRC23, RPLP0, ALDOA, PHB2, UBXN6 and RPS5 (Table 1). Among these co-expressed genes, NDUFA4L2 and SHMT2 had a high co-expression efficient, with Pearson’s Correlation = 0.61 (Fig. 4a). Besides, we also noticed that NDUFA4L2 is a newly biomarker that maybe involved in renal clear cell carcinoma occurrence and progression. To confirm the association between NDUFA4L2 and SHMT2, A498 and ACHN cells were transfected with SHMT2 expression vectors (Fig. 4b-d). In Fig. 4b, we detected SHMT2′s expression in A498 and ACHN cells 24 h after transfection of SHMT2 expression vector or the negative control by western blot. The protein expression of SHMT2 was higher in SHMT2 expression vector group. In Fig. 4c, we found that SHMT2 overexpression could lead to significantly increased NDUFA4L2 expression at mRNA level by qRT-PCR. At the protein levels, NDUFA4L2’s expression was also higher in A498 and ACHN cells 24 h after transfection of SHMT2 expression vector (Fig. 4d).

**Table 1:** Co-expression genes of SHMT2 in kidney cancer by cBioPortal

Correlated Gene	Cytoband	Pearson’s Correlation	Spearman’s Correlation	P-Value	Q-Value
NDUFA4L2	12q13.3	0.61	0.487509244	5.29E − 28	2.13E − 24
TCEA3	1p36.12	0.55	0.536958268	1.13E − 34	2.28E − 30
RACK1	5q35.3	0.54	0.476685233	1.11E − 26	3.19E − 23
HYI	1p34.2	0.51	0.493135371	1.04E − 28	7.01E − 25
LRRC23	12p13.31	0.51	0.471964961	4.03E − 26	8.13E − 23
RPLP0	12q24.23	0.51	0.470753952	5.59E − 26	1.03E − 22
ALDOA	16p11.2	0.5	0.489777334	2.76E − 28	1.39E − 24
PHB2	12p13.31	0.49	0.526836541	3.22E − 33	3.25E − 29
UBXN6	19p13.3	0.46	0.464023674	3.39E − 25	5.70E − 22
RPS5	19q13.43	0.45	0.473084127	2.97E − 26	6.66E − 23

#### Expression levels of NDUFA4L2 in patients with kidney cancer and prognostic analysis

**Fig. 5:**
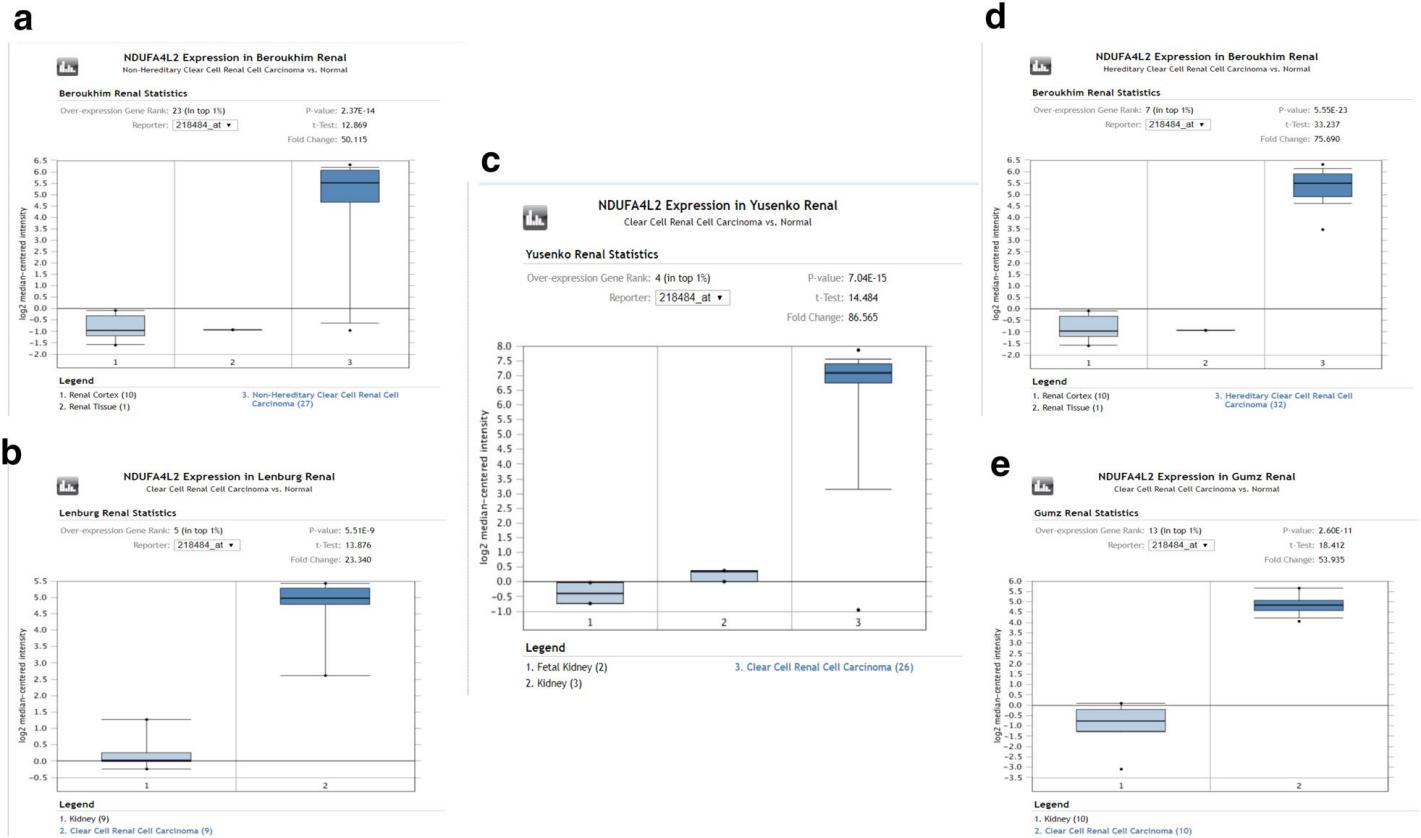
The expression level of NDUFA4L2 mRNA in human different types of kidney cancer, including Clear Cell Renal Cell Carcinoma, Non-Hereditary Clear Cell Renal Cell Carcinoma and Hereditary Clear Cell Renal Cell Carcinoma (ONCOMINE) (**a-e**)

**Fig. 6:**
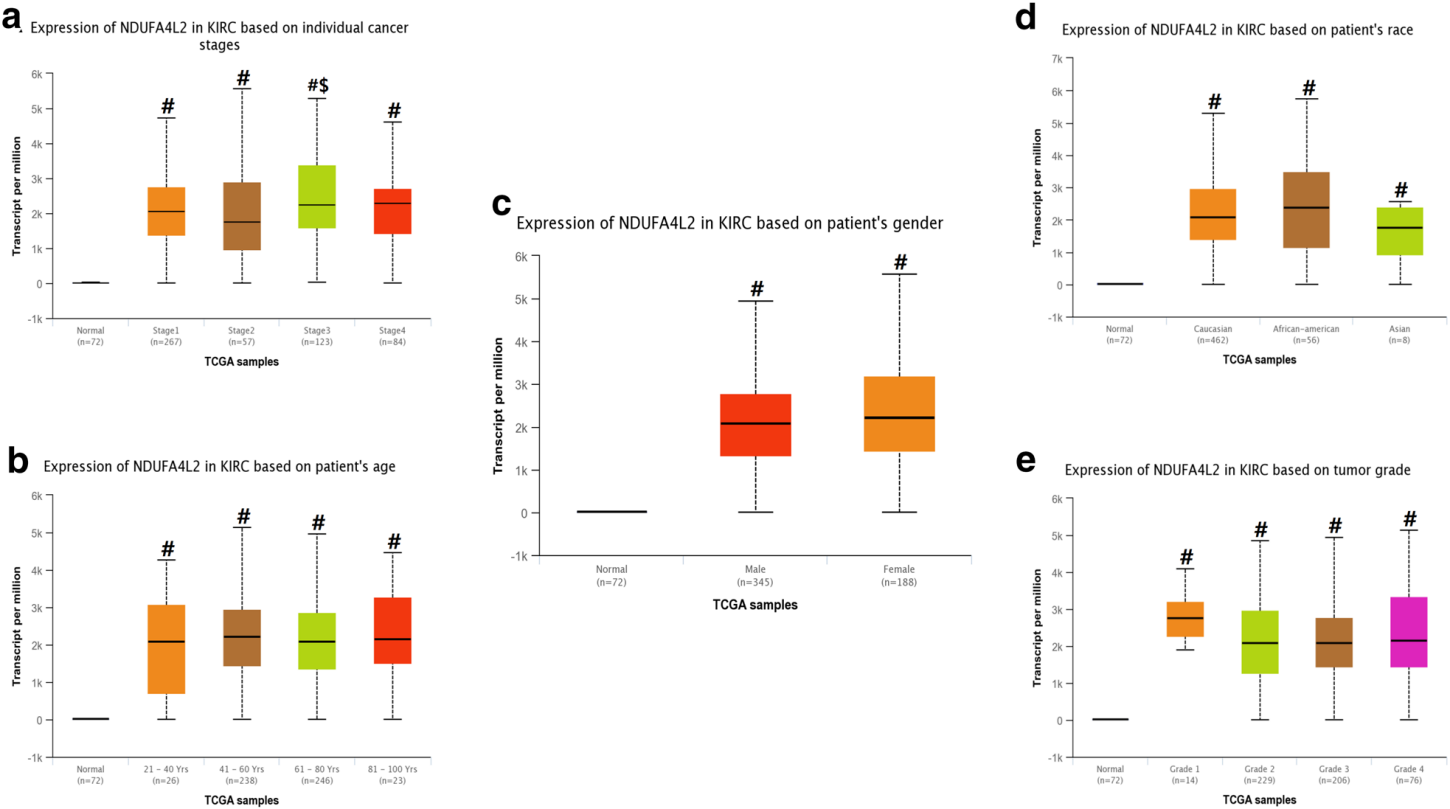
Association between NDUFA4L2 gene expression and clinical pathological parameters in patients with renal clear cell carcinoma (UALCAN). **a** expression of NDUFA4L2 in normal and renal clear cell carcinoma tissues based on cancer stages; **b** expression of SHMT2 in normal and renal clear cell carcinoma tissues based on patients’ age; **c** expression of SHMT2 in normal and renal clear cell carcinoma tissues based on patients’ gender; **d** expression of SHMT2 in normal and renal clear cell carcinoma tissues based on patients’ race; **e** expression of SHMT2 in normal and renal clear cell carcinoma tissues based on tumor grade. #P:compared to normal group; $P: compared to the Stage 2 group

**Fig. 7:**
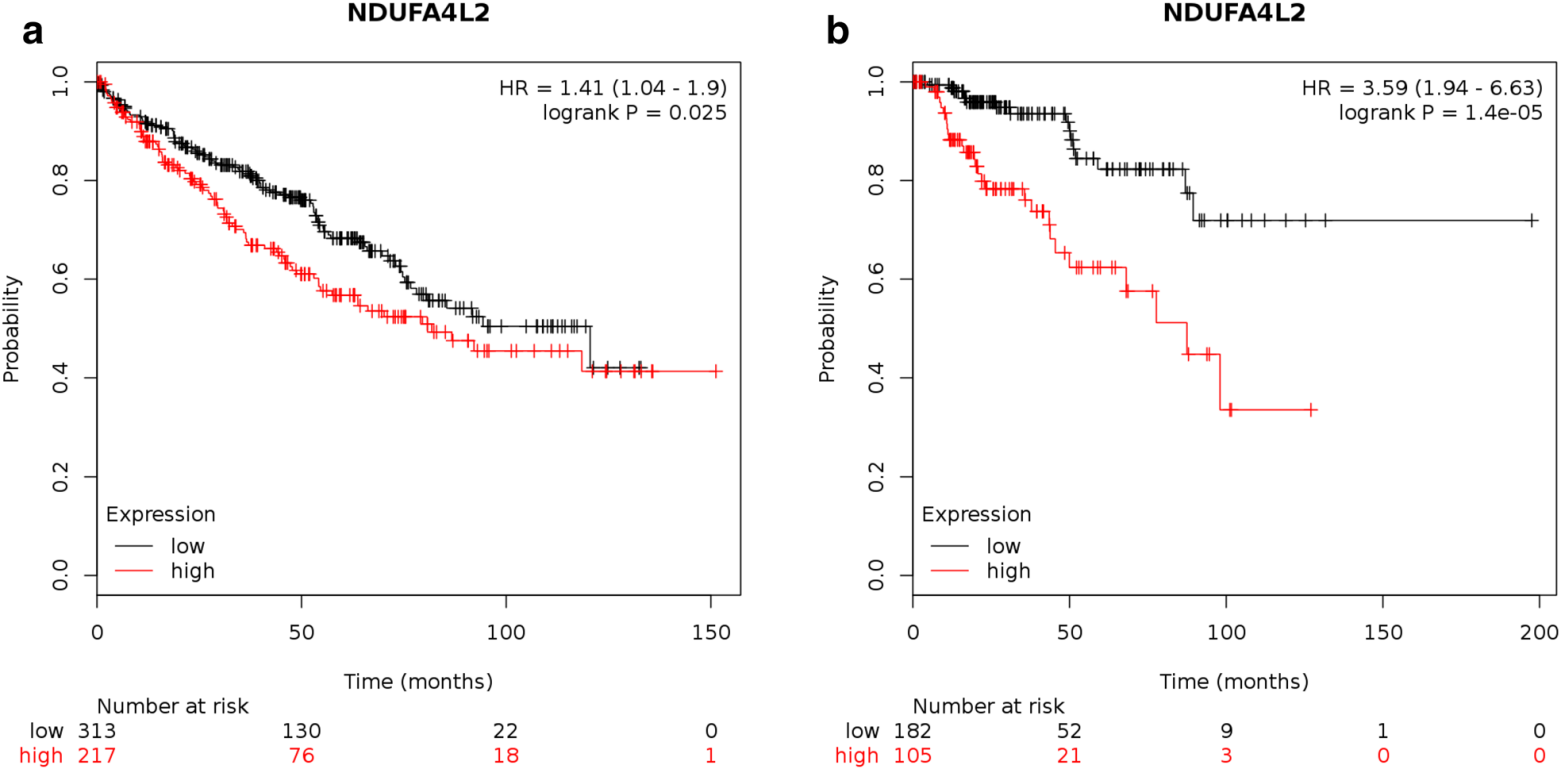
The prognostic value of NDUFA4L2 in patients with kidney cancer **a, b** overexpressed NDUFA4L2 was associated with worse OS in patients with renal clear cell carcinoma and renal papillary cell carcinoma (P < 0.05, respectively)

To further analyze NDUFA4L2 expression in kidney cancer and normal renal tissues, we explored NDUFA4L2’s expression in the above databases. In Oncomine database, NDUFA4L2 was also increased in 5 various kidney cancer datasets, compared to normol tissues (P‑value < 0.05 and Fold change > 2) including Clear Cell Renal Cell Carcinoma, Non-Hereditary Clear Cell Renal Cell Carcinoma and Hereditary Clear Cell Renal Cell Carcinoma (Fig. 5). In UALCAN database, we found that NDUFA4L2 was higher in Stage 3 compared to the Stage 2 and Stage 1 (P < 0.05). However, in respect of gender, races tumor grade and age, there is no significant difference in expression of SHMT2 among different groups (Fig. 6). Like SHMT2, overexpressed NDUFA4L2 also was associated with worse OS in patients with renal clear cell carcinoma and renal papillary cell carcinoma (P < 0.05, respectively) (Fig. 7).

## Discussion

In current research, we found that SHMT2 is overexpressed in different kinds of kidney cancer with those in control samples. Moreover, we also analyzed the correlation of SHMT2 expression with clinicopathological features from patients with renal clear cell carcinoma. As a protein coding gene, SHMT2 also possessed prognostic value for renal clear cell carcinoma, which was also validated by immunohistochemical staining from pathological tissues and healthy tissues. Then Analysis of SHMT2’s co-expressed genes in kidney cancer discovered that NDUFA4L2 was the primary co-expressed gene with SHMT2 in kidney cancer. Through experimental verification, we confirmed that SHMT2 overexpression could lead to significantly increased NDUFA4L2 expression at both mRNA and protein levels in A498 and ACHN cells. After data mining by multiple bioinformatic tools, these results suggested that NDUFA4L2 was also high expressed in various types of kidney cancer and was associated with worse OS in patients with renal clear cell carcinoma, like SHMT2. Our studies provided a novelty biomarker and analyzed its significant clinical relevance in kidney cancer.

SHMT2 encodes the mitochondrial form of a pyridoxal phosphate-dependent enzyme that catalyzes the reversible reaction of serine and tetrahydrofolate to glycine and 5,10-methylene tetrahydrofolate. The encoded product is primarily responsible for glycine synthesis. Current studies showed that SHMT2 may be participated in several kinds of cancers, such as breast cancer [8, 15], glioma [16], intrahepatic cholangiocarcinoma [6] and colorectal cancer [17]. Besides these studies, a recent research also reported that SHMT2 desuccinylation is a pivotal signal in cancer cells to adapt serine metabolic processes for rapid growth. To prevent the tumor growth and proliferation, SIRT5 could be a potential therapeutic target for inhibiting serine catabolism. A meta-analysis also found that SHMT2 and its downstream enzyme MTHFD2 were broadly required for cancer cell proliferation and viability [18]. Through proteomic profiling of breast cancer metabolism, Bernhardt et al. identified the importance of SHMT2 and ASCT2 in breast cancer patients, which could serve as valuable individual prognostic markers and potential targets and may beneficial to personalized breast cancer therapy [8]. In neuroblastoma patient samples, there was a significant correlation between SHMT2 and hypoxia-inducible factor-1 α (HIF-1α), and SHMT2 expression correlated with worse patient prognosis [19]. As for colorectal cancer, Wei et al. demonstrated that SHMT2 is acetylated at K95 in colorectal cancer cells. However, as the major deacetylase in mitochondria, SIRT3 is responsible for SHMT2 deacetylation. Their studies showed that deacetylation of SHMT2 by SIRT3 could enhance colorectal carcinogenesis and was correlated with poorer postoperative overall survival [20]. And a recent publication also showed that SHMT2 is overexpressed in kidney cancers, particulay in the high stage tumors [19]. Our investigations may helpful to further fill in the blank and understand the biological significance of SHMT2 in kidney cancer.

Through co-expression analysis by cBioPortal database, we identified a SHMT2’s co-expressed gene, NDUFA4L2, in kidney cancer. NDUFA4L2’s related pathway included in respiratory electron transport, ATP synthesis by chemiosmotic coupling, and heat production by uncoupling proteins. and metabolism. For hepatocellular carcinoma, Sarathi et al. found that NDUFA4L2, CRHBP and PIGU were main genes with monotonic changes of expression across cancer stages that are expected to be the therapeutic targets [21]. And in non-small cell lung cancer, some researchers also discovered that mitochondrial NDUFA4L2 protein promotes the vitality of lung cancer cells by repressing oxidative stress, suggesting mitochondrial NDUFA4L2 could represent promising targets for therapy [22]. Unlike SHMT2, NDUFA4L2 had been reported to be involved in the occurrence and development of kidney cancer. Lucarelli et al. identified NDUFA4L2 as the most highly expressed gene in renal cancer cells, which is in accordance with our results. Moreover, their studies suggested that NDUFA4L2 played a critical role in regulating angiogenesis and mitophagy in clear cell renal cell carcinoma [23]. In addition, other validation studies also confirmed that application of NDUFA4L2 in clinical practice could also produce useful effect. Although the number of patients were relatively small, Liu et al. found NDUFA4L2 protein expression was found to be higher in renal clear cell carcinoma tissues 81.4% (70/86) than in normal tissues 26.7% (23/86) [24]. Furthermore, NDUFA4L2 overexpressed was reported to be associated with renal clear cell carcinoma malignancy. And its expression was regulated by ELK1 in renal clear cell carcinoma cells [25]. Combined with the above outcomes, we could suppose that SHMT2 and its co-expressed gene NDUFA4L2 may modulate the initiation and development of kidney cancer and influence its prognosis, which may serve as the potential targets for kidney cancer treatment. However, this hypothesis need to be validated by lots of experiments in vivo and vitro.

The limitations of our study included: Firstly, we just validated some findings by polymerase chain reaction and western blot. There are many results need to be verified by multiple experimental methods. Second, the upstream molecule on the roles of SHMT2 in kidney cancer lack of further exploration. It is valued to be explored and verified in the future.

## Conclusion

In summary, SHMT2 and its co-expressed gene NDUFA4L2 were significantly overexpressed in various kinds of kidney cancer. Moreover, high expression of SHMT2 and NDUFA4L2 predicted poor OS in patients with renal clear cell carcinoma. The present study might be benefit for better understanding the clinical significance of SHMT2 and provided a potential therapeutic target for kidney cancer research in the future.

## Data Availability

The datasets analyzed during the current study are available from the corresponding author on reasonable request.

## Abbreviations

SHMT2: Serine Hydroxymethyltransferase 2
ccRCC: Clear cell renal cell carcinoma
NDUFA4L2: NADH dehydrogenase [ubiquinone] 1 alpha sub complex, 4-like 2
GEPIA: Gene Expression Profiling Interactive Analysis
OS: Overall survival
